# Effects of dietary calcium source and quantity on the laying rate, eggshell quality, reproductive tract, liver fat level, and duodenum morphology in Dekalb white laying hens of 90 weeks of age

**DOI:** 10.1016/j.psj.2025.105446

**Published:** 2025-06-16

**Authors:** L. Star, M.A.M. Oosterveer-van der Doelen, F. Molist, R. Gehring, R.R. Santos

**Affiliations:** a Schothorst Feed Research, PO Box 533 8200 AM, Lelystad, the Netherlands; b Veterinary Pharmacotherapy and Pharmacy, Department of Population Health Sciences, Faculty of Veterinary Medicine, Yalelaan 104-106 3584 CM Utrecht University, Utrecht, the Netherlands

**Keywords:** Laying hen, Calcium source, Intestine, Shell gland, Liver fat content

## Abstract

This study evaluated different Ca sources and levels for laying rate, eggshell quality and the reproductive tract, liver fat level, and duodenum morphology of old Dekalb White laying hens. A total of 9,900 hens were randomly assigned to one of 5 experimental diets, with 6 pens per diet and 330 laying hens per pen for 4 wk. The dietary treatments were: TRT1, positive control with hens fed a diet with 39 g/kg Ca from coarse limestone; TRT2, negative control with hens fed a diet with 36 g/kg Ca from coarse limestone; TRT3 with hens fed a diet with 39 g/kg Ca sourced from 75 % coarse plus 25 % fine limestone; TRT4, with hens fed a diet with 39 g/kg Ca from 75 % coarse limestone plus 25 % oyster shells; and TRT5, with hens fed a diet with 39 g/kg Ca from 75 % coarse limestone 25 % eggshells. Egg laying rate, BW, feather scores, and eggshell elasticity and breaking strength were similar in all treatments. Feeding eggshells to the hens (TRT5) resulted in increased duodenum villus height. Serum insulin-like growth factor-1 and serotonin levels were higher in hens from TRT3 compared to TRT5. Growth hormone receptor mRNA expression was upregulated in TRT5 in comparison to TRT1, and tight junction protein claudin-3 was downregulated in all treatments compared to TRT1. The use of eggshells as a Ca source appears to be a sustainable practice for enhancing the production performance of laying hens, possibly due to its ability to preserve intestinal morphology.

## Introduction

Ageing in laying hens causes a decrease in egg laying rate and eggshell quality. This is of special importance when the chickens are kept in production for more than 90 wk. During 23–90 wk of age, eggshell percentage, toughness, and breaking strength decreased by about 7 %, 11 %, and 15 %, respectively (Gloux et al., 2020). Less Ca retention in old laying hens prioritizes Ca for eggshell formation (Gloux et al., 2020). Attia et al. (2020) suggested that laying hens aged 60–72 wk require more Ca during late production to improve laying performance, eggshell quality, and immunological status. Hy-Line Brown laying hens aged 70–80 wk and fed a diet containing 41 g/kg Ca had improved eggshell quality and a reduction in the number of cracked eggs (An et al., 2016). Increased dietary Ca leads to aged laying hens having reduced intestinal Ca absorption (up to 20 %) during 40–70 wk of age (Diana et al., 2021). Not only Ca dietary levels but also Ca homeostasis, which is affected by the hen's age, must be considered. When young (22 wk old) and old (120 wk old) laying hens were subjected to a severe 28-d dietary Ca depletion (from 37.5 to 0.8 g/kg), the older hens were less affected by hypocalcemia because they were already laying fewer eggs and their Ca reserves were smaller than those of the younger hens (Elaroussi et al., 1994). Therefore, increasing the amount of Ca levels in the diet of aged laying hens may not directly result in an improvement in egg laying rate.

The source of dietary Ca also plays an important role in the egg laying rate. For example, coarse limestone shows a positive effect on eggshell quality due to its longer retention in the gizzard and subsequent increased availability of Ca for a greater time compared to fine limestone particles (Guo and Kim, 2012; Jardim Filho et al., 2005; Rao and Roland, 1990). On the other hand, bone resorption is improved by fine limestone and a rapidly available Ca source needed by hens, suggesting a ratio of 75 % coarse and 25 % fine limestone in the diets of aged laying hens (Hervo et al., 2021). Heuser and Norris (1946) suggested oyster shells as an alternative Ca source that has an impact on eggshell strength comparable to limestone. The positive effect of oyster shells has been linked to their large particle size, allowing prolonged Ca availability in the upper digestive tract as observed with coarse limestone (Scott et al., 1971). In laying hens 19–74 wk of age, dietary supplementation with oyster shells resulted in body weight (**BW**) and egg laying rate similar to that in hens fed a conventional diet with coarse limestone as the Ca source (Saunders-Blades et al., 2009). Lee et al. (2021) compared oyster shells with cockles hells and coarse and fine eggshells as dietary Ca sources for 73-wk-old laying hens during 7 wk and observed no differences in eggshell Haugh unit or blood biochemistry, except by a decrease in high-density lipoprotein levels in the hens fed the oyster shells and coarse eggshells. Additionally, coarse eggshells had a positive impact on egg laying performance and tibial mineral density.

Eggshells are considered a Ca source with a higher beneficial effect than oyster shells because eggshells contain not only Ca and P but also up to 2 % crude protein (**CP**) from the eggshell membrane (Islam and Nishibori, 2021; Lertchunhakiat et al., 2016). Eggshells are comparable to limestone regarding their effects on eggshell quality and Ca digestibility, which was not observed when combining limestone with oyster shells (Scheideler, 1998). To improve eggshell quality, Ca metabolism in the eggshell gland must be balanced, which is often dysregulated in aged laying hens (Attia et al., 2020). Moreover, improved ovarian function was linked to enhanced intestinal integrity in old laying hens fed a diet supplemented with Ca (Zhang et al., 2024).

The duodenum is the major site of Ca absorption, and its morphology may be affected by dietary Ca. In broiler chickens, dietary supplementation with an active dicalcium phosphate improved the intestinal villus height (Xing et al., 2020). In contrast, Noruzi et al. (2022) decreased dietary Ca by up to 30 % without affecting jejunum morphology in broiler chickens, but there was no clear indication of the mechanism of action. There is a paucity of information on laying hens. Hepatic lipid metabolism is regulated by Ca signaling, which promotes lipase activity and reduces liver steatosis in mammals (Zhang et al., 2021). High serum Ca levels accompany high cholesterol levels in laying hens experiencing fatty liver syndrome (Harms and Simpson, 1979). Fatty liver hemorrhagic syndrome (**FLHS**) is known to deteriorate egg production and quality in laying hens (Galea, 2011). Moreover, FLHS occurs more frequently in aged laying hens than in younger hens (Dong and Tong, 2019), making the former more vulnerable to production losses and poorer egg quality.

We hypothesize that different Ca sources have the same benefits as conventional coarse limestone. Therefore, in this study we aimed to evaluate different Ca sources on the eggshell quality, reproductive tract, fat content in the liver, and duodenum morphology in aged laying hens. Based on the suggested optimal ratios of coarse and fine limestones (75:25), the same ratio was maintained when testing oyster shells and eggshells. As a negative control, a group was fed a diet with coarse limestone and a decreased Ca level in the diet (36 g/kg instead of 39 g/kg as in all other treatments). A positive control consisted of a diet supplemented with 100 % coarse limestone providing 39 g/kg Ca.

## Materials and methods

### Ethics

The study protocol was approved by the Dutch Central Authority for Scientific Procedures on Animals and the Animal Experiments Committee of Utrecht University (Utrecht, the Netherlands) under registration number AVD24600202114740. All procedures were conducted in full compliance with all relevant legislation.

### Laying hens and housing

The experiment was performed at the trial facility of Schothorst Feed Research (**SFR**), the Netherlands. This facility is mechanically ventilated, and the temperature is controlled by a climate computer. A total of 9,900 86 wk-old Dekalb White laying hens were distributed among 5 treatments, each treatment with 6 replicates in a completely randomized block design. The hens were randomly assigned to 30 experimental units of 330 hens per pen with chopped straw as litter (20.0 m² floor space; 36.8 m² living area). The hens were provided feed and water *ad libitum*.

### Ca sources and diet preparation

The 5 experimental diets were produced in the commercial feed mill of ABZ De Samenwerking (Nijkerk, the Netherlands), and all diets had the same nutrient levels, only the Ca levels or sources were different. Before adding the test Ca sources, the diet contents were analyzed (See Supplementary Table 1), and the Ca supplement and ingredients (e.g., wheat, soybean meal, wheat middlings, oat hulls, monocalcium phosphate, salt, and sodium bircabonate) was adjusted to obtain the experimental dietary Ca levels and to maintain similar nutrient and energy levels. For TRT1, a positive control, the diet contained coarse limestone and 39 g/kg Ca. In TRT2, a negative control, the diet contained coarse limestone and 36 g/kg Ca. The TRT3 diet contained 39 g/kg Ca sourced from 75 % coarse plus 25 % fine limestone. The TRT4 diet contained 39 g/kg Ca sourced from 75 % coarse limestone plus 25 % oyster shells. The TRT5 diet contained 39 g/kg Ca sourced from 75 % coarse limestone plus 25 % eggshells. The oyster shells were provided by the feed mill. The eggshells were provided by Van den Burg Eiproducten B.V. (Waalwijk, the Netherlands) via Interovo Egg Group B.V. (Ochten, the Netherlands).

The coarse limestone, oyster shells, and eggshells had a particle size ranging 2.5–4.0 mm, whereas the fine limestone had a particle size less than 1 mm. Table 1 provides a detailed composition of the diets, each with an energy level of 2,700 kcal/kg. The diets were delivered in bulk and stored in the silos at the SFR layer facility. Approximately every 3-4 wk, a new batch of each diet was delivered. The laying hens were fed these diets when they were 86–100 wk old.

**Table 1:** Composition and calculated nutrient content of the diets.

	PC TRT1	NC TRT2	Fine limestone TRT3	Oyster shells TRT4	Egg shells TRT5
**Ingredients (%)**					
Corn	35.0	35.0	35.0	35.0	35.0
Wheat	18.1	18.5	18.0	18.0	18.3
Soybean meal (>48 % CP)	13.5	12.9	13.5	13.5	13.7
Sunflower seed meal (37 % CP)	12.0	12.0	12.0	12.0	12.0
Wheat middlings	4.2	6.3	4.4	4.7	3.3
Oat hulls	3.1	2.5	3.1	3.0	3.4
Soybean oil	3.0	2.7	3.0	3.0	3.0
Monocalcium phosphate		0.04	0.07	0.07	0.08
Salt		0.18	0.19	0.19	0.19
Sodium bicarbonate		0.22	0.21	0.21	0.21
Premix Lay^1^		0.40	0.40	0.40	0.40
Premix red^2^		0.23	0.23	0.23	0.23
Premix yellow^3^		0.03	0.03	0.03	0.03
Lysine HCl (79 %)		0.05	0.04	0.04	0.04
Methionine L/DL (99 %)		0.14	0.14	0.14	0.14
Choline-Chloride 75 %		0.04	0.04	0.04	0.04
Limestone - coarse	9.73	8.94	7.27	7.24	7.41
Limestone fine	—	—	2.42	—	—
Oyster shells	—	—	—	2.41	—
Eggshells	—	—	—	—	2.47
**Nutrients (g/kg)**					
Energy (kcal/kg)	2,700	2,700	2,700	2,700	2,700
Dry matter		890	892	892	892
Ash		123	131	130	133
Crude protein		165	165	165	165
Crude fat	53.5	50.9	53.5	53.5	53.7
Crude fiber	45.0	45.0	45.0	45.0	45.0
Starch	337	342	337	337	337
Ca	39.0	36.0	39.0	39.0	39.0
P	4.15	4.24	4.16	4.17	4.12
K	7.43	7.53	7.44	7.46	7.37
Na	1.50	1.50	1.50	1.50	1.50
Cl	1.80	1.80	1.80	1.80	1.80
dEB	205	208	205	206	204
SID LYS	6.60	6.60	6.60	6.60	6.60
SID MET	3.98	3.96	3.98	3.98	3.99
SID *M* + *C*	6.27	6.27	6.27	6.27	6.27
SID THR	4.92	4.90	4.92	4.92	4.93
SID TRP	1.70	1.71	1.71	1.71	1.70
SID VAL	6.75	6.74	6.75	6.75	6.75
SID ILE	5.86	5.83	5.86	5.86	5.87
SID ARG	10.1	10.1	10.1	10.1	10.1

TRT1: Positive Control (PC), diet with a coarse limestone as a Ca source at a level of 39 g/kg;.

TRT2: Negative Control (NC), diet with a coarse limestone as a Ca source at a level of 36 g/kg;.

TRT3: 75 % coarse limestone + 25 % fine limestone as Ca source at a level of 39 g/kg;.

TRT4: 75 % coarse limestone + 25 % fine oyster shells as Ca source at a level of 39 g/kg;.

TRT5: 75 % coarse limestone + 25 % eggshells as Ca source at a level of 39 g/kg.

### Measurements

*Production Performance, Eggshell Quality, and Feather Condition.* The BW of the hens was recorded weekly per pen. The egg laying rate was recorded during 4-wk periods, i.e., from wk 86 to 90, wk 91 to 95, and wk 96 to 100, and reported as the percentage of the number of eggs produced by the number of hens in each pen. Eggshell quality was determined by evaluating elasticity and breaking strength. Thirty eggs were randomly collected from each pen at wk 90, 95, and 100 of age. The Institute for Quality Measurement in Eggs (IKE, Amersfoort, the Netherlands) performed the measurements. The feather condition of 5 hens per pen was determined at 90 and 100 wks of age. The feather condition score described by Bilčik and Keeling (1999) and modified by Uitdehaag et al. (2008) was used. Scores, varying from 0 (intact feathers, no injuries or scratches) to 5 (completely denuded area), were applied to each of 5 body parts (head, neck, back, rump, and belly). Only these body parts of the hen were scored because they are related to feather-pecking behavior (Uitdehaag et al., 2008). Scores for the different parts were added and averaged per pen for analysis of treatment effects.

*Tissue Sampling.* At 90 wk of age, 1 hen per pen was euthanized with carbon dioxide, and the BW was recorded. From each euthanized hen, 5 mL blood was collected into serum tubes, and the serum was harvested by centrifugation (15 min at 1,500 rpm), and stored at −20°C until further biochemical analysis. Subsequently, the liver, small intestine, ovary, oviduct, and eggshell gland were collected and weighed. From each collected ovary, follicles greater than 12 mm (hierarchical follicles, i.e., ∼3–5 days to ovulation; Nie et al., 2022) were counted. The liver fat content was determined by ether extraction. The small intestine was weighed immediately after collection (full) and again after careful manual removal of its content (empty).

*Histological Analysis.* After weighing the intestine, a 1-cm section of the duodenum was fixed in 4 % paraformaldehyde. The sections were stained with periodic acid–Schiff, counterstained with hematoxylin stain, and scanned using a NanoZoomer-XR (Hamamatsu Photonics KK, Hamamatsu, Japan). The scanned slides were viewed through the viewer software NDP.view2 version 2.27.25 (Hamamatsu Photonic KK) and analyzed by the software NDP.analyze (Hamamatsu Photonics KK). The villus height (**VH**), crypt depth (**CD**), and villus area (**VA**) of at least 10 villi per intestinal segment from each euthanized bird were measured, and the VH:CD ratio was calculated (Santos et al., 2014).

*mRNA Expression.* Samples of the duodenum (1 cm) and eggshell gland (1 cm) were collected for mRNA expression of markers related to function and Ca absorption and deposition. The collected tissues were preserved in RNALater (Sigma-Aldrich, St. Louis, MI, USA). The RNA was extracted using the SV Total RNA Isolation System (Promega, Madison, WI, USA) according to the manufacturer’s instructions. Primers (Table 2) were commercial products (Eurogentec, Maastricht, the Netherlands). The primers used were selected based on specificity and efficiency by qPCR analysis of dilution series of pooled cDNA at a temperature gradient (55–65°C) for primer annealing and subsequent melting curve analysis. qPCR was performed using the MyIQ single-color real-time PCR detection system (Bio-Rad) and MyiQ System Software Version 1.0.410 (Bio Rad Laboratories Inc., USA). The data were analyzed using the efficiency-corrected DeltaDelta-Ct method (Pfaffl, 2001). The fold-change values of the genes of interest were normalized using the geometric mean of the fold-change values of the housekeeping genes for hypoxanthine-guanine phosphoribosyl transferase (**HPRT**) and glyceraldehyde-3-phosphate dehydrogenase (**GAPDH**). The mRNA expression levels of proteins involved in transepithelial Ca transport (plasma membrane Ca ATPase – PMCA1b), eggshell calcification (ovocalyxin-32 – OCX32 and ovocalyxin-36 – OCX36), tight junction proteins (claudin-1 – CLDN1, claudin-3 – CLDN3, and claudin-5 – CLDN5), growth (growth hormone receptor – GHR and insulin-like growth factor-1 receptor – IGF1R), and apoptosis (caspase-9 – CASP9 and B-cell lymphoma 2 – BCL2) were measured.

**Table 2:** Primers used for the mRNA quantification of genes of interest (GOI) and housekeeping genes (HKG) in the duodenum and eggshell gland of the laying hens.

Genes	NCBI accession number	Primer sequence	Annealing T°	Reference
*HKG*				
GAPDH	NM_204305	F: GTGTGCCAACCCCCAATGTCTCT R: GCAGCAGCCTTCACTACCCTCT	57	Milanova et al., 2016
HPRT	NM_204848.1	F: CGTTGCTGTCTCTACTTAAGCAG R: GATATCCCACACTTCGAGGAG	65	Santos et al., 2019
*GOI*				
PMCA1b	XM_046906440.1	F: TTCAGGTACTCATGTGATGGAAGG R: CAGCCCCAAGCAAGGTAAAG	60	Liu et al., 2023
OCX32	NM_204534.4	F: CCAAGAAGAGGACCACAGATT R: CAACAGCATTGTCCTTCCTTATC	60	Zhu et al., 2020
OCX36	XM_025142254.1	F: CAAGCTGATCTCTGGCTTACTG R: GGAAGGTGTATGGCTGGATATG	60	Zhu et al., 2020
CLDN1	AY750897	F: GACTCGCTGCTTAAGCTGGA R: AAATCTGGTGTTAACGGGTG	60	Park et al., 2011
CLDN3	NM_204202	F: AGCCCTCCATCTCAGCAG R: TTCTCCGCCAGACTCTCC	60	Ozden et al., 2010
CLDN5	NM_204201	F: GTCCCGCTCTGCTGGTTC R: GCCCTATCTCCCGCTTCTGG	60	Ozden et al., 2010
GHR	AB075215	F: TTACTTCAACACATCCTACACC R: TCATAATCTCTTCCCATCTTCA	60	Ni et al., 2007
IGF1R	BQ037565	F: GTACTTCAGTGCTTCGGATGTG R: CTTCTTCAGAGTTGGAGGTGCT	60	Ni et al., 2007
CASP9	XM_424580.6	F: GGAACATTACGCCCGTTCTG R: ACGATGTCTGACACCCGAAGT	60	Reno et al., 2022
BCL2	NM_205339.2	F: TCGTCGCCTTCTTCGAGTTC R: CATCCCATCCTCCGTTGTCC	60	Reno et al., 2022

GAPDH: glyceraldehyde 3-phosphate dehydrogenase; HPRT: hypoxanthine-guanine phosphoribosyl transferase; PMCA1b: Plasma membrane Ca ATPase; OCX32: ovocalyxin-32; OCX36: ovocalyxin-36; CLDN1: claudin-1; CLDN3, claudin-3; CLDN5: claudin-5; GHR: growth hormone receptor; IGF1R: insulin-like growth factor-1 receptor; CASP9: caspase-9; BCL2: B-cell lymphoma 2.

### Statistical analyses

The pen was the experimental unit for all data. The experimental data were analyzed using ANOVA (GenStat Version 22.0, 2022; Hemel Hempstead, UK). Treatment means were compared with Tukey’s post hoc test. Values with *p* ≤ 0.05 were considered statistically significant. The p-value and SEM (standard error of the mean) are given per response parameter.

## Results

### Production performance, eggshell quality, and feather condition

**Figure 1:**
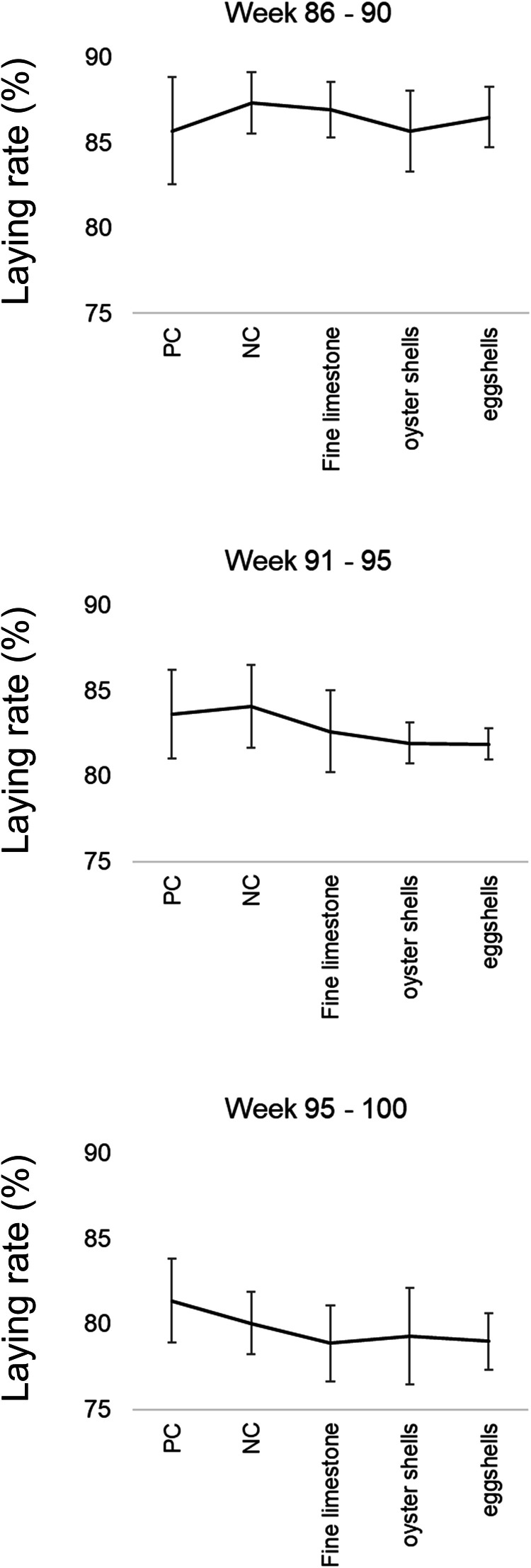
Mean (± SD) laying rate (%) of the laying hens in the period of 86 – 90 weeks of age, 91 – 95 weeks of age, and 96 – 100 weeks of age after being fed diets differing in Ca source.

No significant differences were observed in laying rate among the dietary treatments or the sampling periods (Fig. 1). Additionally, BW, eggshell elasticity and breaking strength, and feather scoring were not significantly affected by dietary treatment (Table 3).

**Table 3:** Mean BW (g), eggshell elasticity (N/s), breaking strength (N), and feather scoring of laying hens fed diets differing in Ca source at 90, 95, and 100 weeks of age.

TRT	Description	BW (g)	Elasticity	Breaking strength	Feather so
		(g)	N/s	N	scoring
Week 90					
1	PC	1821	594	39.8	2.55
2	NC	1799	573	39.2	2.61
3	Fine limestone	1818	590	38.2	2.93
4	Oyster shells	1829	569	37.1	3.12
5	Eggshells	1828	607	38.8	2.99
	*P-value*	0.20	0.06	0.11	0.37
	*SEM*	9.51	9.20	0.69	0.24
Week 95					
1	PC	1824	601	36.6	NA
2	NC	1781	578	37.4	NA
3	Fine limestone	1816	593	38.4	NA
4	Oyster shells	1824	578	38.0	NA
5	Eggshells	1815	579	37.4	NA
	*P-value*	0.06	0.22	0.61	*
	*SEM*	9.94	8.48	0.79	*
Week 100					
1	PC	1814	578	35.1	2.93
2	NC	1793	576	35.0	2.63
3	Fine limestone	1809	599	34.7	2.57
4	Oyster shells	1801	580	34.7	2.93
5	Eggshells	1793	584	35.7	2.87
	*P-value*	0.27	0.87	0.82	0.72
	*SEM*	16.48	16.48	0.66	0.24

TRT1: Positive Control (PC), diet with a coarse limestone as a Ca source at a level of 39 g/kg;.

TRT2: Negative Control (NC), diet with a coarse limestone as a Ca source at a level of 36 g/kg;.

TRT3: 75 % coarse limestone + 25 % fine limestone as Ca source at a level of 39 g/kg;.

TRT4: 75 % coarse limestone + 25 % fine oyster shells as Ca source at a level of 39 g/kg;.

TRT5: 75 % coarse limestone + 25 % eggshells as Ca source at a level of 39 g/kg.

Feather scores were reported on weeks 90 and 100 only.

NA: Not applicable.

### Liver, small intestine, and reproductive tract

In wk 90, liver weight was not affected by dietary treatment. The liver fat content in TRT1 hens (PC) did not differ from the other treatments, but it was significantly less (*P* < 0.02) in TRT2 hens (NC) than in TRT5 hens (eggshells) (Table 4). Dietary treatment did not affect the relative weight of the small intestine (full and empty), oviduct, eggshell gland, and reproductive tract. The relative weight of the ovaries from TRT5 hens (eggshells) was significantly greater (*P* < 0.02) than the weight of the ovaries from TRT4 hens (oyster shells). The relative weight of the ovaries from TRT4 hens (oyster shells) was also significantly less (*P* < 0.02) than the weight of the ovaries from TRT2 hens (NC). The level and source of dietary Ca did not affect the number of ovarian follicles larger than 12 mm (Table 5).

**Table 4:** Mean liver relative weight (% of BW) and liver fat content (g/kg) of 90-weeks-old laying hens fed diets differing in Ca source.

TRT	Description	Relative liver weight (% BW)			Liver fat content (g/kg)	
1	PC	2.52		71.29		ab
2	NC	2.53		57.45		a
3	Fine limestone	2.64		72.88		ab
4	Oyster shells	2.72		89.87		ab
5	Eggshells	2.70		96.84		b
	*P-value*	0.91		0.02		
	*SEM*	0.14		11.61		

TRT1: Positive Control (PC), diet with a coarse limestone as a Ca source at a level of 39 g/kg;.

TRT2: Negative Control (NC), diet with a coarse limestone as a Ca source at a level of 36 g/kg;.

TRT3: 75 % coarse limestone + 25 % fine limestone as Ca source at a level of 39 g/kg;.

TRT4: 75 % coarse limestone + 25 % fine oyster shells as Ca source at a level of 39 g/kg;.

TRT5: 75 % coarse limestone + 25 % eggshells as Ca source at a level of 39 g/kg.

^a,b^ Values without a common superscript per column differ significantly (*P* ≤ 0.05).

**Table 5:** Mean relative weight (% of BW) of intestine (full and empty), ovary, oviduct, eggshell gland, and complete reproductive tract, and the mean number of large yellow follicles per ovary of 90-weeks-old laying hens fed diets differing in Ca source.

TRT	Description	Intestine full (% BW)	Intestine empty (% BW)	Ovary (% BW)		Oviduct (% BW)	Eggshell gland (% BW)	Reproductive tract (% BW)	Follicle >12 mm (number)
1	PC	4.78	2.85	2.63	ab	8.52	2.80	13.94	4.33
2	NC	4.76	3.14	3.08	b	8.93	3.01	15.03	5.67
3	Fine limestone	4.45	2.97	2.68	ab	8.73	2.72	14.73	5.50
4	Oyster shells	4.83	2.93	2.21	a	7.51	2.73	11.92	5.17
5	Eggshells	4.70	3.05	3.00	b	8.63	2.63	13.95	6.00
	*P-value*	0.66	0.38	0.02		0.23	0.90	0.08	0.06
	*SEM*	0.19	0.11	0.18		0.45	0.29	0.75	0.38

TRT1: Positive Control (PC), diet with a coarse limestone as a Ca source at a level of 39 g/kg;.

TRT2: Negative Control (NC), diet with a coarse limestone as a Ca source at a level of 36 g/kg;.

TRT3: 75 % coarse limestone + 25 % fine limestone as Ca source at a level of 39 g/kg;.

TRT4: 75 % coarse limestone + 25 % fine oyster shells as Ca source at a level of 39 g/kg;.

TRT5: 75 % coarse limestone + 25 % eggshells as Ca source at a level of 39 g/kg.

^a,b^ Values without a common superscript per column differ significantly (*P* ≤ 0.05).

### Duodenum histomorphometry

The duodenum VH of TRT5 hens (eggshells) was significantly higher (*P* = 0.01) compared to all other treatments, whereas the VH of TRT3 hens (fine limestone) was significantly lower (*P* = 0.01) compared to TRT2 hens (NC). The duodenum VA of TRT5 hens (eggshells) was significantly higher (*P* = 0.03) compared to TRT2 hens (NC) (Table 6).

**Table 6:** Mean villus height (VH; µm), crypt depth (CD; µm), villus height:crypt depth ratio (VH:CD), and villus area (VA; mm^2^) in the duodenum of 90-weeks-old laying hens fed diets differing in Ca source.

TRT	Description	VH (µm)		CD (µm)	VH:CD (µm:µm)	VA (mm^2^)	
1	PC	1111	ab	263	4.41	207	ab
2	NC	1251	b	256	4.94	188	a
3	Fine limestone	1135	ab	303	3.87	227	ab
4	Oyster shells	992	a	314	3.29	229	ab
5	Eggshells	1443	c	258	5.73	319	b
	*P-value*	0.01		0.42	0.20	0.03	
	*SEM*	72.9		20.2	0.48	26.6	

TRT1: Positive Control (PC), diet with a coarse limestone as a Ca source at a level of 39 g/kg;.

TRT2: Negative Control (NC), diet with a coarse limestone as a Ca source at a level of 36 g/kg;.

TRT3: 75 % coarse limestone + 25 % fine limestone as Ca source at a level of 39 g/kg;.

TRT4: 75 % coarse limestone + 25 % fine oyster shells as Ca source at a level of 39 g/kg;.

TRT5: 75 % coarse limestone + 25 % eggshells as Ca source at a level of 39 g/kg.

^a-c^ Values without a common superscript per column differ significantly (*P* ≤ 0.05).

### Serum analysis

**Figure 2:**
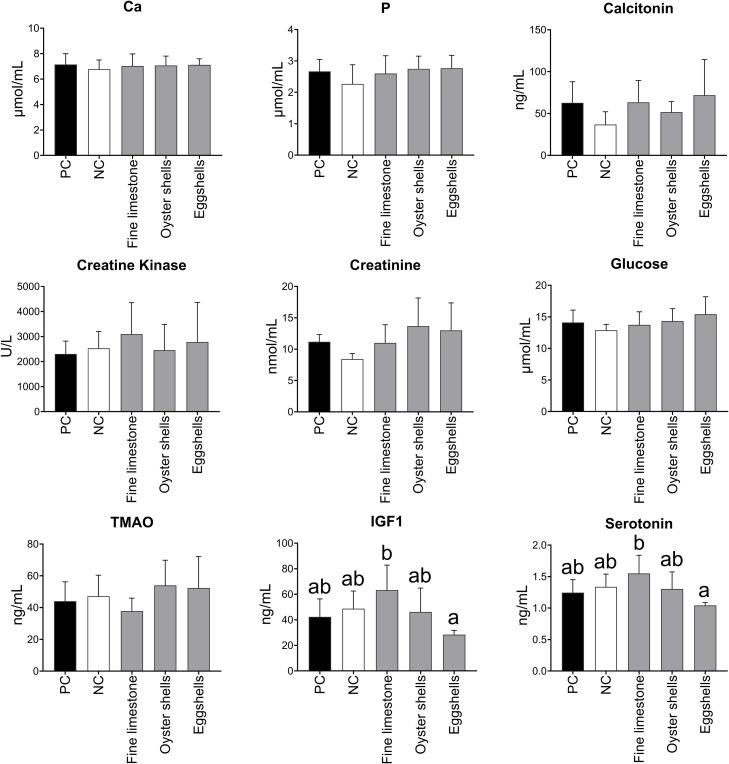
Mean (± SD) serum levels of Ca (mmol/L), P (mmol/L), calcitonin (ng/mL), creatine kinase (U/L), creatinine (µmol/L), glucose (mmol/L), TMAO (ng/mL), IGF1 (ng/mL), and serotonin (ng/mL) of 90-week-old laying hens fed diets differing in Ca source. a,b Values without a common superscript per column differ significantly (*P* ≤ 0.05).

None of the dietary treatments affected serum levels of Ca, P, calcitonin, creatine kinase, creatinine, glucose, and trimethylamine N-oxide (TMAO). However, serum levels of IGF-1 and serotonin were significantly highest (*P* < 0.05) in TRT5 hens (eggshells) (Fig. 2).

### mRNA expression

**Figure 3:**
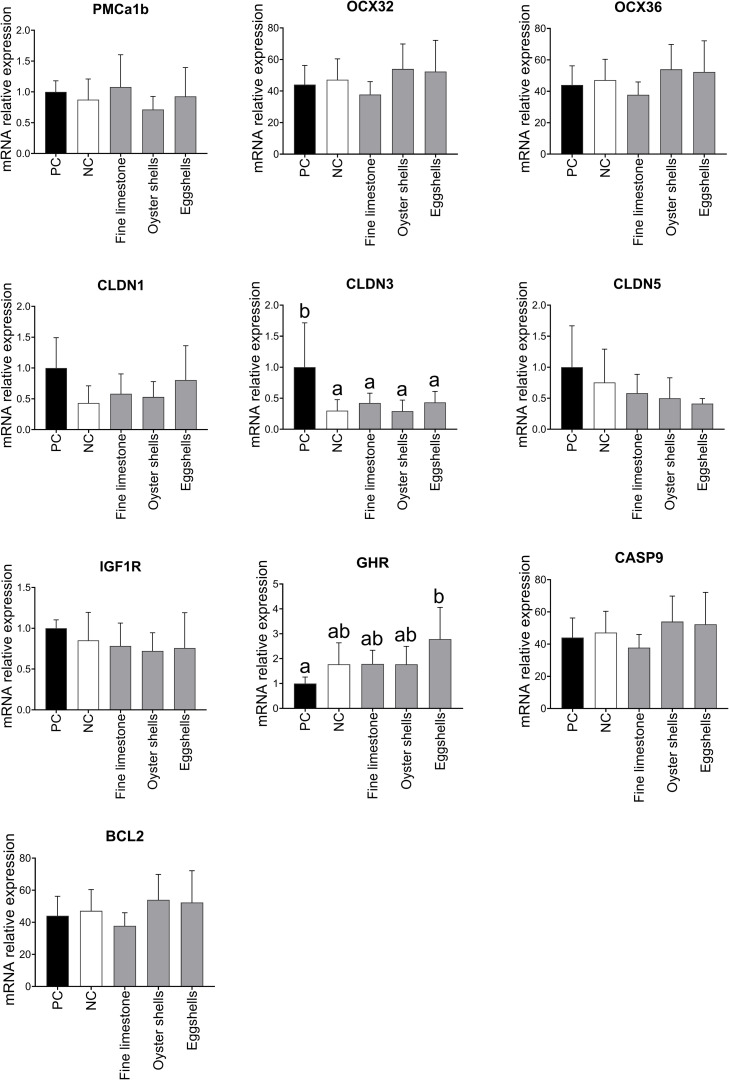
Mean (± SD) mRNA relative expression of PMCa1b, OCX32, OCX36, CLDN1, CLDN3, CLDN5, IGF1R, GHR, CASP9, and BCL2 in the eggshell gland of 90-week-old laying hens fed diets differing in Ca source. ^a,b^ Values without a common superscript per column differ significantly (*P* ≤ 0.05).

Dietary treatment did not affect mRNA expression of the selected proteins in the duodenum of the laying hens (Supplementary Fig. 1). In the eggshell gland, there was a downregulation (*P* < 0.05) of CLDN3 in all treatments when compared to TRT1 (PC). Hens in TRT5 (eggshells) presented an up-regulation (*P* < 0.05) of GHR in their eggshell gland when compared to the TRT1 (PC) (Fig. 3).

## Discussion

In our study, decreasing dietary Ca from 39 g/kg to 36 g/kg did not impair egg laying rate, BW, feather condition, eggshell quality (elasticity and breaking strength), the number of large ovarian follicles, weight of the reproductive tract and intestine, duodenum morphology, nor serum Ca levels. The source of Ca, however, played a role in the weight of the ovaries, intestinal morphometry, serum levels of IGF-1, and the relative expression of GHR.

Although a decreased capacity of intestinal Ca absorption takes place in aged laying hens, it has been proposed that a decrease in dietary Ca will result in a compensatory response that leads to increased Ca retention to maintain blood Ca homeostasis, resulting in decreased Ca content in the eggshell (Jiang et al., 2023). In our study, serum levels of Ca and P and eggshell elasticity and breaking strength were unchanged when dietary Ca levels were decreased. However, levels of Ca in the eggshell were not measured. Fat metabolism can be regulated by Ca, where low Ca diets will induce fat accumulation and the opposite effect is observed with high dietary Ca (Zemel et al., 2000). Roland et al. (1985) found that a decrease of Ca from 37.5 to 10 g/kg in the diet resulted in increased feed intake and subsequent fat accumulation in the liver. It is noteworthy that Roland et al. (1985) reported that fat represented about 20 % of the liver weight, whereas in our study the difference in Ca levels was very low (3 g) and fat represented a maximum of 5 % of the liver eight. The liver fat content in hens receiving 36 g/kg sourced from coarse limestone differed from hens receiving 39 g/kg Ca sourced from coarse limestone plus eggshells. However, the difference of these two treatments cannot be properly explained because it might be related to Ca level or to Ca source. The post-mortem analysis did not diagnose fatty liver syndrome.

Dietary supplementation with eggshells as a Ca source resulted in a significant increase in ovarian weight compared to the treatment with oyster shells. The increase in ovarian weight, however, did not affect the number of large follicles. Unfortunately, the population of small ovarian follicles was not recorded in our study. Guinotte et al. (1991) demonstrated that oyster shells have lower solubility than eggshells, due to the higher porosity and bioavailability of eggshells compared to the more compact structure of oyster shells, which makes Ca absorption difficult. The greater ovarian weight in hens receiving 36 g/kg sourced from coarse limestone compared to hens receiving 39 g/kg Ca sourced from coarse limestone plus oyster shells could be related to Ca level and source, but this cannot be determined by our experimental design.

Ca plays a crucial role in the maintenance of intestinal integrity and morphology, as shown in a study with broiler chickens (Xing et al., 2020). Besides minerals, eggshells are rich in hundreds of matrix proteins, including antimicrobial proteins and peptides that are known for their ability to support intestinal morphology (Liu et al., 2022; Moreau et al., 2022). Further research on the composition of eggshells, including the stability of proteins and peptides after processing, may explain our findings that duodenum VH increased when the hens were fed coarse limestone plus eggshells.

Serum levels of Ca, P, calcitonin, creatine kinase, creatinine, glucose, and TMAO were unaffected by dietary treatment, whereas IGF-1 and serotonin serum levels were significantly higher in laying hens fed a diet with coarse plus fine limestone compared to those fed a diet with coarse limestone plus eggshells). Wu et al. (2024) described the role of IGF-1 in growth and bone development in laying hens, in which the increased serum levels of this endocrine hormone may indicate a decrease in growth.

Elevated serum serotonin levels may be an indicator of a decrease in the risks of feather pecking or other stressful behaviors (Son et al., 2025), which were not observed in our trial. In mammals, the serotonin-Ca axis is well studied, where the neurotransmitter serotonin acts to maintain circulating Ca concentrations, stimulating Ca mobilization from bones (Connelly et al., 2021; Weaver et al., 2016). In vitro studies with chicken enterocytes showed that serotonin stimulates the release of Ca from intracellular reserves (Chang and Much, 1990). The shorter time of Ca availability in fine limestone particles compared to eggshells likely stimulated Ca mobilization in our study’s hens, as evidenced by the increased serum serotonin levels and similar eggshell quality in the hens with eggshells in their diet compared to the other treatments.

Feeding the laying hens with eggshells significantly increased the expression of GHR in their eggshell glands in comparison to hens in the positive control group. The eggshell gland is a site for GH activity (Ni et al., 2007), and increased expression of GHR is linked to the synthesis of minerals needed for eggshell formation (Hrabia et al., 2013). However, this increased upregulation was not consistent with an increase in the weight of reproductive organs in our study, nor did it affect eggshell elasticity or breaking strength. mRNA expression of PMCA1b, OCX32, OCX36, CLDN1, CLDN3, CLDN5, IGF1R, CASP9, and BCL2 was not affected by dietary treatment, indicating that the amount of Ca in the diet and/or the Ca source were not responsible for the modulation of the eggshell gland function in the 90-wk-old laying hens.

In conclusion, eggshells can effectively replace 25 % of the coarse limestone in the diet of 90-wk-old laying hens without negatively affecting egg laying rate and eggshell elasticity and breaking strength. Additionally, eggshells in the diet can increase duodenum VH. Because eggshells are a sustainable source of Ca, our results indicate their use as a promising waste management strategy, especially in the diet for old laying hens. Our study also indicates that aged laying hens can cope with a decrease in dietary Ca from 39 to 36 g/kg without adverse effects on egg laying rate, eggshell elasticity and breaking strength, duodenum morphology, circulating levels of minerals, and the expression of proteins for eggshell formation and mineralization in the eggshell gland. Additional studies combining decreased dietary Ca levels with various Ca sources are needed to confirm our findings. For instance, further research evaluating the supplementation of diets with eggshells and decreased Ca levels in the diet could confirm this beneficial effect for aged laying hens. A long-term dietary intervention is needed to corroborate our present results.

## Declaration of competing interest

the authors declare no conflict of interest.

## Appendix Supplementary materials

Image, application 1

Image, application 2

**Supplementary Figure 1.** Mean (± SD) mRNA relative expression of PMCa1b, OCX32, OCX36, CLDN1, CLDN3, CLDN5, IGF1R, GHR, CASP9, and BCL2 in the duodenum of 90-week-old laying hens fed diets differing in Ca source. Image, application 3
