# Supplementary material for: Effects of dietary calcium source and quantity on the laying rate, eggshell quality, reproductive tract, liver fat level, and duodenum morphology in Dekalb white laying hens of 90 weeks of age
Source: Poult Sci. 2025 Jun 16;104(9):105446. doi: 10.1016/j.psj.2025.105446 (PMC12221496; doi:10.1016/j.psj.2025.105446)
Supplement: Supplementary file 1 [file mmc1.pdf]

Proof-Reading-Service.com
PhD theses, journal papers, books and other professional documents

Proof-Reading-Service.com Ltd, Devonshire Business Centre, Works Road, Letchworth Garden City, Hertfordshire, SG6 1GJ, United Kingdom
Office phone: +44(0)20 31 500 431
E-mail: enquiries@proof-reading-service.com
Internet: http://www.proof-reading-service.com
VAT registration number: 911 4788 21
Company registration number: 8391405

12 June 2025

To whom it may concern,

RE: Proof-Reading-Service.com Editorial Certification

This is to confirm that the document described below has been submitted to Proof-Reading-Service.com for editing and proofreading.

We certify that the editor has corrected the document, ensured consistency of the spelling, grammar and punctuation, and checked the format of the sub-headings, bibliographical references, tables, figures etc. The editor has further checked that the document is formatted according to the style guide supplied by the author. If no style guide was supplied, the editor has corrected the references in accordance with the style that appeared to be prevalent in the document and imposed internal consistency, at least, on the format.

It is up to the author to accept, reject or respond to any changes, corrections, suggestions and recommendations made by the editor. This often involves the need to add or complete bibliographical references and respond to any comments made by the editor, in particular regarding clarification of the text or the need for further information or explanation.

We are one of the largest proofreading and editing services worldwide for research documents, covering all academic areas including Engineering, Medicine, Physical and Biological Sciences, Social Sciences, Economics, Law, Management and the Humanities. All our editors are native English speakers and educated at least to Master's degree level (many hold a PhD) with extensive university and scientific editorial experience.

**Document title:**	**Effect of Ca source and level on eggshell quality and the relation with reproductive tract, liver fat level and duodenum morphology in Dekalb white laying hens of 90 weeks of age**
**Author(s):**	**L. Star, M.A.M. Oosterveer-van der Doelen, F. Molist, R. Gehring, and R.R. Santos**
**Format:**	**American English**
**Style guide:**	**Poultry Science at https://www.sciencedirect.com/journal/poultry-science/publish/guide-for-authors**
