# Supplementary material for: Effects of dietary calcium source and quantity on the laying rate, eggshell quality, reproductive tract, liver fat level, and duodenum morphology in Dekalb white laying hens of 90 weeks of age
Source: Poult Sci. 2025 Jun 16;104(9):105446. doi: 10.1016/j.psj.2025.105446 (PMC12221496; doi:10.1016/j.psj.2025.105446)
Supplement: Supplementary file 2 [file mmc2.docx]

**Supplementary Table 1.** Calcium levels in the coarse and fine limestones, oyster shells, and egg shells used in the diets.

|  | **Coarse limestone** | **Fine limestone** | **Oyster shells** | **Egg shells** |
| --- | --- | --- | --- | --- |
| Ca (g/kg) | 359.99 | 380.75 | 361.55 | 361.76 |
